# Performance of the Thies Clima 3D Stereo Disdrometer: Evaluation during Rain and Snow Events

**DOI:** 10.3390/s24051562

**Published:** 2024-02-28

**Authors:** Sabina Angeloni, Elisa Adirosi, Alessandro Bracci, Mario Montopoli, Luca Baldini

**Affiliations:** 1 National Research Council of Italy, Institute of Atmospheric Sciences and Climate (CNR-ISAC), 00133 Rome, Italy; sabina.angeloni@artov.isac.cnr.it (S.A.); elisa.adirosi@artov.isac.cnr.it (E.A.); m.montopoli@isac.cnr.it (M.M.); 2 National Research Council of Italy, Institute of Atmospheric Sciences and Climate (CNR-ISAC), 40129 Bologna, Italy; a.bracci@isac.cnr.it; 3 Department of Physical and Chemical Sciences (DSFC), University of L’Aquila, 67100 L’Aquila, Italy

**Keywords:** laser disdrometer, imaging disdrometer, rain microphysics, snow

## Abstract

Imaging disdrometers are widely used in field campaigns to provide information on the shape of hydrometeors, together with the diameter and the fall velocity, which can be used to derive information on the shape–size relations of hydrometeors. However, due to their higher price compared to laser disdrometers, their use is limited to scientific research purposes. The 3D stereo (3DS) is a commercial imaging disdrometer recently made available by Thies Clima and on which there are currently no scientific studies in the literature. The most innovative feature of the 3DS is its ability in capturing images of the particles passing through the measurement volume, crucial to provide an accurate classification of hydrometeors based on information about their shape, especially in the case of solid precipitation. In this paper. the performance of the new device is analyzed by comparing 3DS with the Laser Precipitation Monitor (LPM) from the same manufacturer, which is a known laser disdrometer used in many research works. The data used in this paper were obtained from measurements of the two instruments carried out at the Casale Calore site in L’Aquila during the CORE-LAQ (Combined Observations of Radar Experiments in L’Aquila) campaign. The objective of the comparison analysis is to analyze the differences between the two disdrometers in terms of hydrometeor classification, number and falling speed of particles, precipitation intensity, and total cumulative precipitation on an event basis. As regards the classification of precipitation, the two instruments are in excellent agreement in identifying rain and snow; greater differences are observed in the case of particles in mixed phase (rain and snow) or frozen phase (hail). Due to the different measurement area of the two disdrometers, the 3DS generally detects more particles than the LPM. The performance differences also depend on the size of the hydrometeors and are more significant in the case of small particles, i.e., D < 1 mm. In the case of rain events, the two instruments are in agreement with respect to the terminal velocity in still air predicted by the Gunn and Kinzer model for drops with a diameter of less than 3 mm, while, for larger particles, terminal velocity is underestimated by both the disdrometers. The agreement between the two instruments in terms of total cumulative precipitation per event is very good. Regarding the 3DS ability to capture images of hydrometeors, the raw data provide, each minute, from one to four images of single particles and information on their size and type. Their number and coarse resolution make them suitable to support only qualitative analysis of the shape of precipitating particles.

## 1. Introduction

Precipitation is one of the most important meteorological phenomena that affect everyday life. Precise and accurate measurement of precipitation is essential for many applications. For instance, precipitation is a fundamental input for understanding the global water cycle, the management of water resources and transport infrastructures, planning agricultural operation, and predicting events with high societal impact like landslides and flooding [1]. Precipitation can be measured by means of a large variety of different devices [2]. Studies comparing different rainfall measurement devices and methods have highlighted the challenges in obtaining accurate precipitation estimates [2,3,4]. Rain gauges are the most traditional instruments for providing local precipitation data and yield accurate and direct measurement of rain accumulation (in mm) in a given amount of time. Nowadays, the use of disdrometers in precipitation measurement is increasing thanks to their capability to provide the microphysical structure of hydrometeors in terms of drop size distribution and other properties rather than in terms of precipitation accumulation as provided by raingauges [5]. There are several types of disdrometers. At the moment, thanks to its good trade-off between cost and accuracy of estimates, and to its ease of management, the most popular one is the laser disdrometer. It estimates, with a high temporal resolution, typically 1 min [6], relevant characteristics, such as diameter and fall velocity of particles passing through a measurement area defined by a laser beam. Among the laser disdrometers, this study considers the Laser Precipitation Monitor (LPM) manufactured by Thies Clima GmbH, Germany. Several studies have been carried out in order to compare the performance of LPM with respect to other instruments for estimation of rainfall and microphysical parameters [7,8,9] and for a wide range of applications in addition to the quantitative estimation of precipitation (e.g., the estimation of kinetic energy of rain [10], the development of remote sensing retrieval techniques [11], and the classification of solid precipitation [12]). Also, laboratory experiments [13] have been carried out to evaluate its accuracy in windy conditions. Therefore, LPM can be considered a reference instrument, well known both in terms of points of strength and limitations.

Another category of widely used disdrometers is that of optical imaging sensors. In general, they use a photographic sensor, operating in the visible band, to capture images of each hydrometeor crossing a sensing volume. An advantage of these instruments, with respect to laser disdrometers, is their ability to also provide information on the shape of hydrometeors in addition to their diameter and fall velocity. This ability has been used to derive information on the shape–size relations of drops, fundamental to develop and characterize the behavior of dual-polarization weather radar algorithms for quantitative rainfall estimation [14,15] and for investigating shapes of ice particles characterized by high variability.

The 2D video disdrometer (2DVD) [16,17], commercially available from Joanneum Research Forschungsgesellschaft mbH, Austria, has been adopted in many campaigns and has been used in research regarding the shape of hydrometeors. It uses two orthogonally oriented horizontal line-scan cameras to take measurements of shadows due to precipitation particles passing through a 10 cm × 10 cm area illuminated by two internal lamps. Since the viewing planes of the two cameras are separated by around 6–7 mm, it is possible to measure the fall speed from the time interval between arrival times of a drop in the two planes. Images of the two cameras are collected every 18 microseconds and are combined to produce a two-dimensional image of the precipitation particle for each camera with a resolution of ∼0.2195 mm.

In addition to 2DVD, two other optical disdrometers have been commonly used in the literature to investigate ice particles’ shapes, namely Precipitation Imaging Particles (PIP) and the Multi Angle Snowflakes Camera (MASC).

PIP [18,19] is composed of two parts: a single high-speed video camera and a light source to backlight the precipitation particles that pass through the open sampling volume. The camera, 2 m far from the light source, records 380 images of 640×480 pixels in a second with a pixel size of 0.1 mm by 0.1 mm that are then compressed to achieve an effective pixel size of 0.1 and 0.2 mm in the horizontal and vertical planes, respectively. Its focal plane is at 1.33 cm from the camera. MASC [20] uses three CCD cameras separated by 36° in azimuth with a focal plane at 10 cm from the lens. The cameras look at a virtual sampling volume, defined by the intersection of the 35 mm fields of view and the 10 mm field depths of the cameras. A particle passes through the field of view of a pair of near-infrared sensors triggering the three cameras and the corresponding flashes opposite to the camera to illuminate the particle. The three cameras have a pixel size of 33.5 μm × 33.5 μm, although particles larger than 0.1 mm are retained [21].

Another optical imaging gauge is the high-speed optical disdrometer (HOD), composed of a high-speed CCD camera, an LED light with a diffuser, and a digital fiber-optic sensing unit [22]. The camera and the light are installed at a distance of 160 cm, and the sensor is installed between the camera and the light source at the focal plane of 60 cm from the camera. The sensor captures particle images at 1000 frames per second with a resolution of 1024×1024 pixels. The measurement volume is defined by the vertical size and horizontal camera view frame (70 mm × 70 mm). The other dimension of resolution volume is defined by the sensor beam width (5.25 mm centered around the focal plane) instead of the field depth of the camera. The sensor is also used to trigger the camera.

Practical problems of optical disdrometers are the purchase cost and maintenance difficulties and, in some cases, commercial availability. The performance of these instruments is also critically dependent on the image processing software adopted, especially if the shape of hydrometeors is the main target of research [21].

Thies Clima has recently made available a commercial optical disdrometer, the 3D stereo disdrometer (3DS). The purpose of this article is to evaluate the 3DS, which, as far as we know, has never been used for scientific purposes before, by comparing it with LPM, the rather popular laser disdrometer from the same manufacturer. The most innovative feature of the new disdrometer lies in capturing images of particles passing through the measurement volume defined by cameras, while a laser disdrometer measures the size and fall velocity of a raindrop that, passing through a surface defined by a laser beam, interrupts the light and causes a reduction in the intensity of the received signal. Imaging capability of 3DS is of crucial importance in providing an accurate classification of hydrometeors, especially in the case of solid precipitation. The 3DS disdrometer is expected to be helpful both regarding rain and snowfall rate, mitigating the difficulties related to the microphysical variability of solid hydrometeors [23]. The study reported in this article aims to highlight the difference in performance of the two Thies Clima disdrometers (i.e., LPM and 3DS) in terms of hydrometeor classification, spectrograms, number of particles, particle fall speed, and rain rate. Given the different measurement principles, differences in performance are expected between the two disdrometers.

This article is organized as follows. Section 2 deals with the description of the main characteristics of the two disdrometers. Section 3 explains the experimental datasets used in the study, and Section 4 describes the comparison and validation approach. Section 5 presents the main results obtained by comparing the 3DS and LPM precipitation outputs. The imaging capabilities of 3DS are also discussed in this section. Finally, Section 6 points out the main findings, which are summarized and commented on.

## 2. Instruments

### 2.1. Laser Precipitation Monitor

The Laser Precipitation Monitor is a laser optical device used for the measurement of diameter and fall velocity of hydrometeors. From such measurements, it is possible to classify different types of precipitation, such as drizzle, rain, hail, snow, and mixed precipitation, quantify precipitation in a given time interval, and derive size and velocity joint distribution. All measuring values are available for the user via an RS 485/422 interface. The instrument body consists of a central unit with emitter diodes and a receiver, connected by brackets arranged on each side of the beam. LPM uses an infrared (780 nm) laser-optical beam covering a nominal sampling area of 45.6 cm^2^, resulting in 228 mm length and 20 mm width, and with a thickness of 0.75 mm. The operating principle is based on the reduction in the receiving electrical signal, into which the laser emission has been converted, because of the passage of hydrometeors through the laser beam. Diameters and fall velocities of hydrometeors are obtained with the aid of this signal reduction by evaluating the obscuration amplitude and the duration of the voltage reduction, respectively. The measured data at one-minute resolution are processed by software provided by the manufacturer in order to check the plausibility of the measurements. The details of this processing are not fully disclosed. The count of falling particles is stored in a 22 diameter × 20 falling velocity matrix, called spectrogram. The diameter classes start from a minimum diameter of 0.125 mm, while the last class includes all the diameters above 8 mm. The classes have a variable width. Fall velocity classes range from 0 to 20 m·s^−1^ (see Table A1 and Table A3 in Appendix A for the definition of diameter and velocity class intervals). LPM telegram also comprises the intensity, amount, and type of precipitation. The latter is determined from the statistical proportion of all particles referring to diameter and velocity according to [24]. The identification is improved by temperature data: precipitation with a temperature of above 9 °C is automatically accepted as liquid, with the exception of hail and soft hail, and with a temperature below −4 °C as solid. In the temperature range between, all forms of precipitation might occur. In addition, the LPM data telegrams make available to users other parameters, which are useful for evaluating data quality. Further technical information can be found in [25].

### 2.2. 3D Stereo Disdrometer

The 3DS is a stereo camera device for measuring all the hydrometeors (drizzle, rain, snow, hail, and mixed precipitation) and acquiring the intensity, amount, and type of precipitation, together with the particle spectrum (i.e., the distribution of the particles over the class binning). Furthermore, it is also able to identify non-hydrometeors. All measuring values are available for the user via Ethernet, RS485 interface, and files on SD card. The instrument is equipped with integrated heating in the camera module housing and a temperature sensor. The instrument body consists of a light source and a stereo camera system that are connected by bracket. The focal plane is at 25 cm from the cameras, which have a sampling rate of 50 frames per second. The working principle is based on the reduction in the intensity of the light reaching the cameras due to the passage of falling particles through the measurement volume. The 3DS used in this paper has a horizontal (i.e., parallel to the ground) measuring area equal to 15,242.90 mm^2^ (personal communication from Thies Clima); however, this value depends on the calibration of the device and can change a bit for the other 3DS. It is worth noting that this area is about three times that of the LPM. As a consequence, 3DS detects more particles than LPM. Diameters are derived from the shadow area reaching the cameras and the position of the particles within the sampling volume. Fall velocities are obtained by evaluating the movement of the particles in a fixed time interval. As for the LPM, the measured raw data at one-minute resolution are processed by the manufacturer’s software. Unlike the LPM, in the 3DS precipitation spectrum, the number of particles is corrected by their detection probability. Moreover, in the 3DS, the diameter and fall velocity classes can be configured by the user, complying with a maximum diameter value of 40 mm and a maximum fall velocity value of 20 m s^−1^ (every class limit will be clipped at this value). The user can set the minimum width and the number of diameter and fall velocity classes. A straightforward relation is used by the software to increment the width as a function of diameter and fall velocity. In this paper, the diameter size and falling velocity are divided into 22 and 20 classes (as for the LPM) ranging from 0 to 12.3 mm and 0 to 28 m s^−1^, respectively (see Table A2 and Table A4 in Appendix A for the classes adopted in this experiment, which actually do not coincide exactly with those of LPM).

With respect to LPM, 3DS is expected to take advantage of the imaging capabilities. The ability to capture images of the particles through the cameras should provide a more accurate classification of hydrometeors at least in the classes defined by WMO weather codes. The classification of the precipitation type is carried out based on shape, size, velocity, and presence of water in particles. The images are captured when a particle passes through the region within ±1.5 cm with respect to the focal point of the cameras. Although the 3DS is able to capture images of all the particles within its measuring volume, in the output telegram, only images of single particles are reported. In particular, each telegram can contain up to 4 images. Each image represents a single hydrometeor in a 12 × 12 matrix. Information on the dimensions and fall velocity of the hydrometeor in the images are also provided. Further technical details can be found in [26].

## 3. Experimental Setup

### 3.1. Validation Site

**Figure 1:**
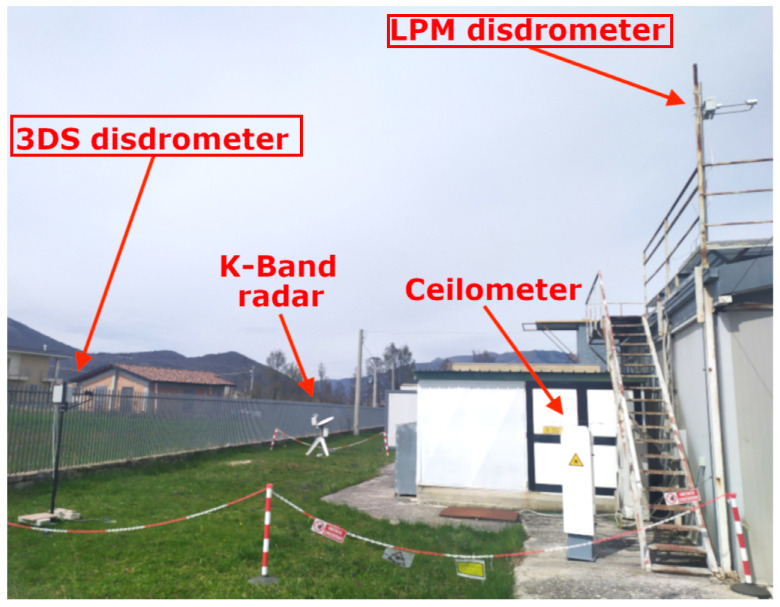
Deployment of LPM and 3DS at the Casale Calore site with other instruments used in the CORE-LAQ campaign (adapted from [27], courtesy of the authors).

Data analyzed in this study are collected in Casale Calore (42.383081 N, 13.314806 E, 683 m ASL, an instrumented site in Central Italy equipped with a pair of co-located LPM and 3DS (Figure 1) and managed by the University of L’Aquila and Center of Excellence in Telesensing of Environment and Model Prediction of Severe Events (CETEMPS). LPM and 3DS are operated for a campaign called CORE-LAQ (Combined Observations of Radar Experiments in L’AQuila) conducted with the Institute of Atmospheric Sciences and Climate (ISAC) of the National Research Council of Italy (CNR) to test precipitation and Doppler parameter retrievals from radar profilers operating at W- and K-band frequencies (94 GHz and 24 GHz, respectively) [27]. The observatory is located in a valley with a temperate climate, with warm summers and without a dry season (Köppen–Geiger-type Cfb). On average, annual precipitation is 620 mm, autumn being the rainiest season, with chances of snow episodes between December and March. From December 2022 to July 2023, CORE-LAQ provided the opportunity to assess in both rain and snow the 3DS of the Italian National Antarctic Research Program (PNRA) before its installation at the Italian Mario Zucchelli research station in Terranova Bay, Antarctica. The 3DS was shipped to Antarctica on July 2023, while the LPM continues routine acquisitions at the Casale Calore site.

It should be noted that LPM in Casale Calore is part of the GID (the Italian group for disdrometry) network, a community-driven initiative to federate disdrometers belonging to different actors that at the moment cover most of the Italian territory [28].

### 3.2. Datasets

The analysis is carried out on precipitation events that occurred from 13 December 2022 to 19 July 2023 at the Casale Calore site. A rain event is considered to last at least 60 min, with a maximum of 30 consecutive no-rain minutes. A similar definition applies to snow events. In the case of rain events, if the rainfall amount is less than 1.5 mm, the event is discarded. Furthermore, the 10 min preceding and following the event itself are also considered part of the event, in order to avoid information loss deriving from any unlikely but possible time shifts of few minutes between the two devices. The selection of the rain events is driven by LPM disdrometer rainfall rate data and classification, while, for snow events, the 3DS classification is taken as a reference; the reasons for this choice will be clear in the sequel. In order to better discriminate between rain and snow events, we consider the ambient temperature detected by the 3DS so that snow events are always associated with a temperature below 4 degrees. The analysis counts a total of 37 rain events and 6 snow events. The maximum and average values for the duration of rainfall events are 1382 and 346 min, respectively; as for the mean rain rate value per event, the maximum is about 5 mm h^−1^ and the average is equal to 1.6 mm h^−1^. More detailed information on the events can be found in Appendix B (see Table A5).

## 4. Comparison and Validation Approach

The comparison between LPM and 3DS is carried out based on the spectrograms of diameters and fall velocities, the precipitation classification, and the rain rate provided by the Thies Clima software, ver. 1.1220, which are part of the considered 1 min data telegrams. The 1 min spectrograms are expressed through matrices

(1) $$n^{X}\left(i,j\right),\;i=1,\ldots ,N_{D}^{X},\;j=1,\ldots ,N_{V}^{X},$$

where superscript *X* identifies the disdrometer and can be LPM or 3DSD. $N_{D}^{X}$ and $N_{V}^{X}$ are the number of diameter and fall velocity bins, respectively, that are different for the two disdrometers (see Table A1 and Table A2).

Drop size distributions, which are maybe the most important expected output for hydrological applications and for developing remote sensing algorithms, are not provided by the manufacturers of disdrometers but are derived by users through additional processing that can imply filtering of spurious hydrometeors, e.g., using, a filter based on a priori fixed mask (such as in [4,28,29]) or chosen adaptively (as in [30]). In addition, LPM data are sometimes corrected using other reference instruments as in [12].

Although evaluation of DSD retrieval methods is beyond the scope of this paper, some implications of LPM and 3DS measurements for DSD retrievals are discussed. Particular attention is focused on the fall velocity of hydrometeors. In rain, the fall velocity diameter relations from [24] experiments, confirmed also by more recent experiments [14], can be considered, on average, as representative of the fall behavior of raindrops at least for diameters smaller than 6 mm, although an example of noticeable deviation is reported in [31]. For larger drops, a slightly decreasing velocity with respect to the fit to [24] was found [14]. In this article, in rain, a formula based on [24], such as [32]

(2) $$v\left(D,0\right)=9.65-10.3e^{-0.6D},$$

can be used for reference at a height of 0 m above sea level (ASL), while modification for a generic height *h* (in m) to take into account air density is according to [33]

(3) $$v\left(D,h\right)=\left(9.65-10.3e^{-0.6D}\right){\left(\frac{\rho (0)}{\rho (h)}\right)}^{0.37+0.025D},$$

where ρ(0) and $\rho \left(h\right)$ in kg·m^−3^ are the air density at sea level and at height *h*, respectively, that can be assumed according to the International Standard Atmosphere Model [34]. Formula (3) will be used in this study, with h=683 m corresponding to the altitude ASL of Casale Calore, and it will be referred to as GK.

Ice particles can take different shape, density, and habits that make their velocity–size relation quite variable, although several laws are available for some reference hydrometeors. This fact allows for a hydrometeor classification based on diameter–velocity pairs [12], and an instrumental bias in velocity estimation will result in classification error.

The second part of validation deals with the evaluation of the detection capability of 3DS with respect to LPM and the comparison of the total accumulated precipitation per event (in mm) between the two disdrometers in order to point out the performance of 3DS in providing accurate rainfall measurements. This part concerns rain events only.

The detection capability is analyzed by means of contingency tables together with POD (Probability of Detection), FAR (False Alarm Ratio), and ACC (Accuracy) indices. Each contingency table contains the following parameters: the number of ‘hits’ minutes Hi, when precipitation is detected by both the 3DS and the LPM; the number of ‘false alarm’ minutes Fa, when precipitation is detected by the 3DS but not by the LPM; the number of ‘missing’ minutes Mi, when precipitation is detected by the LPM but not by the 3DS; and the number of ‘reject’ minutes Re, when both disdrometers do not detect precipitation. POD, FAR, and ACC are statistical indices that6 can be computed from contingency tables. The POD index measures the probability that 3DS correctly detects precipitation when precipitation is actually present (i.e., detected by the LPM). The FAR index is calculated as the ratio between the number of no-rain minutes wrongly categorized as rain minutes (false positives) and the total number of actual no-rain minutes.

The ACC index shows how close measurements by 3DS are to the LPM:

(4) $$\mathrm{POD}=\frac{Hi}{Hi+Mi}$$

(5) $$\mathrm{FAR}=\frac{Fa}{Hi+Fa}$$

(6) $$\mathrm{ACC}=\frac{Hi+Re}{Hi+Fa+Mi+Re}.$$

The rainfall accumulation per event is computed by considering the value provided by the Thies Clima software for all the rainy minutes between the beginning and the end of the event itself. Then, a quantitative comparison is conducted by calculating the correlation coefficient, RMSE (Root Mean Square Error), NMAE (Normalized Mean Absolute Error), and NB (Normalized Bias). The correlation coefficient measures the degree to which the two sets of data are linearly related. It assumes values in the range from −1 to +1, where ±1 indicates the strongest possible agreement and 0 the strongest possible disagreement. Root Mean Square Error allows to evaluate how concentrated the data are around the line of best fit,

(7) $$\mathrm{RMSE}=\sqrt{\bar{{\left(Y-X\right)}^{2}}},$$

where *Y* is the vector representing 3DS data and *X* is the vector of LPM data. The Normalized Mean Absolute Error is a normalization of the mean absolute error (MAE):

(8) $$\mathrm{NMAE}=\frac{MAE}{\bar{Y}},\;\mathrm{where}\;\mathrm{MAE}=\frac{1}{N}\sum_{i=1}^{N}\left|Y_{i}-X_{i}\right|,$$

*N* being the sample size. The Normalized Bias provides information on the quality of the difference of the two datasets: negative NB values indicate an underestimation of 3DS with respect to LPM, while positive NB indicates overestimation:

(9) $$\mathrm{NB}=\frac{\sum_{i=1}^{N}Y_{i}}{\sum_{i=1}^{N}X_{i}}-1.$$

A qualitative analysis of 3DS images is finally conducted for liquid and solid hydrometeors. It should be noted that dependency of LPM measurements from horizontal wind has been noticed in experiments [35] and justified by the Computational Fluid Dynamics simulations [13], pointing out the possible dependency of the performance LPM from wind intensity and also by direction because of the non-symmetric shape of the instrument. Such effects are expected to be more pronounced in light rain and in snow. In fact, for snow, some authors prefer not to rely on LPM measurement if horizontal wind exceeds a given threshold (it is 7 m·s^−1^ in [36]). The geometry of 3DS, also non-symmetric, enables thinking about the influence of horizontal wind, although it is expected to be different from that of LPM. In this paper, we do not consider the likely different effect of wind on the two devices, which deserves different analysis methods, such as laboratory experiments.

## 5. Results

### 5.1. Comparison of Classifications and Analysis of Spectrograms

The first part of this section deals with the comparison between LPM and 3DS classification of particles. The mask applied by the manufacturer for classification is not known, nor the minimum number of hydrometeors belonging to a specific class that must be detected in a minute for providing a given classification. However, both disdrometers provide information on precipitation type by means of 13 precipitation classes as defined by the WMO (Word Meteorological Organization) codes (i.e., Table code 4677). The detection analysis is applied to four precipitation types, obtained by grouping different WMO classes with similar characteristics. In particular we considered the following precipitation types: rain (SYNOP codes 51, 53, 55, 58, 59, 61, 63, and 65), rain with snow (SYNOP codes 68 and 69), snow (SYNOP codes 71, 73, 75, and 77), and hail (SYNOP codes 87, 88, 89, and 90) [37].

**Figure 2:**
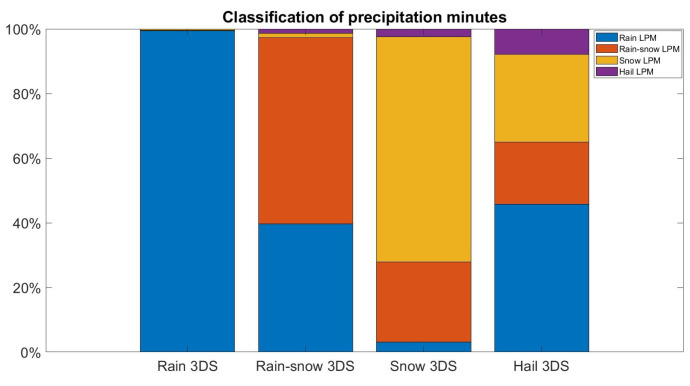
Barplot of the comparison between the classifications of the two disdrometers in percentage. Each bar relates to a 3DS class.

Figure 2 shows the comparison between the number of minutes detected for each class by the two disdrometers. Each bar relates to a 3DS class. The barplot is in terms of percentage of the total number of minutes related to the 3DS classification considered in order to better visualize the relationships between the classifications given that the minutes of rain are many more than the minutes in which other types of precipitation occur. Table 1 shows the contingency tables obtained by considering all the minutes in which precipitation was detected by at least one of the two instruments. The related statistical indices POD, FAR, and ACC are provided in Table 2. Figure 2 and Table 1 and Table 2 show that the agreement between the two tools is excellent as regards the classification of rain, for which the best results are obtained in terms of error indices. A high probability of detection characterizes the snow class, for which, however, the FAR index is high due to the minutes that are not detected as snow by the LPM. The accuracy index is optimal for all the classes; however, it is not indicative in the case of hail and snow mixed with rain due to the lower frequency of this type of event. Major differences in the classification occur for rain mixed with snow and hail, as highlighted by the POD and FAR indices.

In this article, we focus on the analysis of rain and snow events. Based on what has just been discussed, we will use the LPM classification to define rainfall events. As highlighted, for the rain class, the two instruments are in good agreement with each other. For snow events, we will use the 3DS classification, which is potentially more reliable since it takes advantage of image analysis.

**Figure 3:**
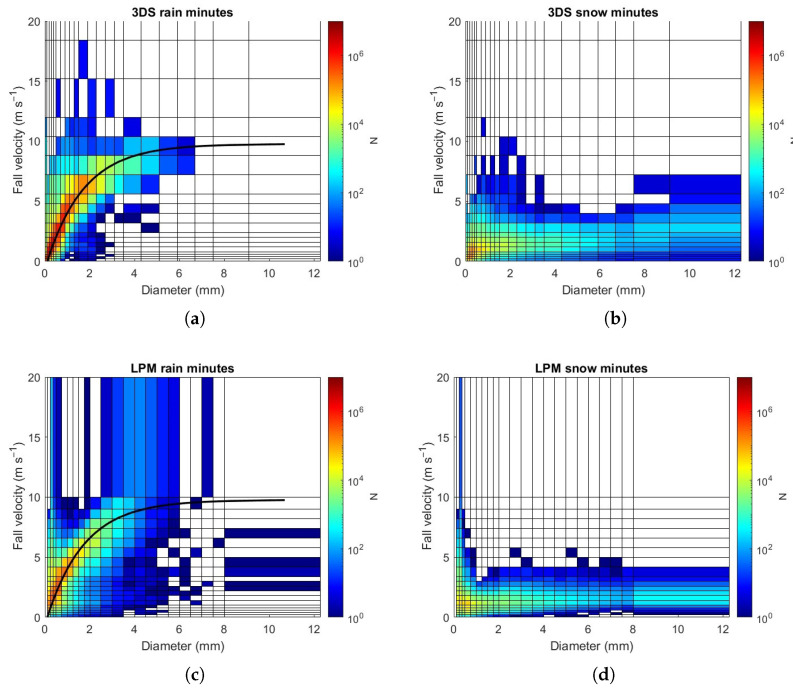
Spectrograms of (**a**,**c**) rainy minutes; (**b**,**d**) snowy minutes. Top line refers to 3DS, the bottom line to LPM. Colors represent the number of particles detected for each bin. Black curves in rain spectrograms represent the reference fall velocity provided by Formula (3).

The spectrograms of Figure 3 concern all the events recorded in Casale Calore and consider the diameter and velocity ranges common to both the disdrometers. The first row presents rain events and the lower row concerns snow events. In particular, the spectrograms relating to rain (or snow) events show the data of minutes associated with rain (or snow) according to the classification of the LPM (or 3DS).

This figure enables an initial comparison of the instruments. The 2D spectrograms in rain show that most of the drops are within an interval close to the curve (3). The 3DS can detect higher velocities, whereas LPM has an upper class with a lower bound of 10 m·s^−1^. In particular, the 3DS has more classes in the range of 10 to 20 m·s^−1^ than the LPM, so it can provide more detailed information about fall velocities. Additionally, the fraction of raindrops with small diameters and high fall velocity seems lower than detected by the LPM, which could be charged to artifact or a physical behavior as well [38,39] This behavior is also present in the LPM snow spectrogram, likely due to artifacts or wrong classification of snow events by the instrument. In the case of rain events, it is worth noting that no particles with diameter greater than 6.7 mm are detected by 3DS.

A thorough comparison of the properties derived from spectrograms will be developed in the next subsections.

### 5.2. Comparison between LPM and 3DS Fall Velocity Measurements

**Figure 4:**
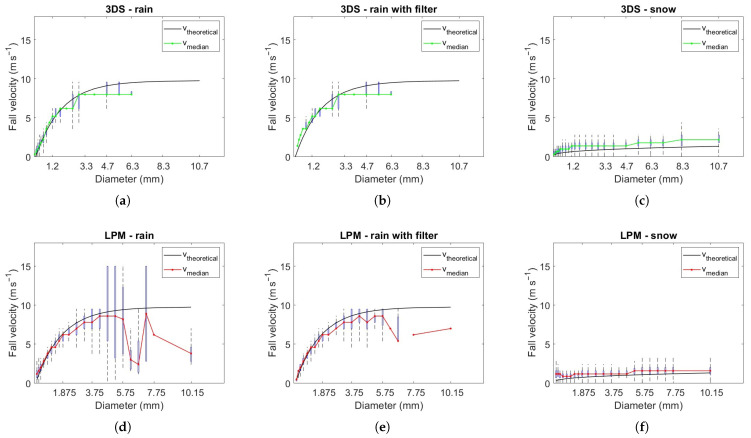
Statistics on terminal fall velocity with respect to diameter class (median value, first and third quartiles, and maximum and minimum) for (**a**,**d**) rain events; (**b**,**e**) rain events with filter; (**c**,**f**) snow events. Top line refers to 3DS, the bottom line to LPM. Black curves represent the theoretical speeds provided by Formula (3) for raindrops, LH for snow particles.

This section examines the performance of the two disdrometers in terms of fall velocity measurements by analyzing and comparing the raw data collected by LPM and 3DS during the common minutes in rain or snow events. With the aid of the spectrograms related to all the precipitation events, statistics on the terminal drop fall speed as a function of the diameter class are provided as shown in Figure 4.

Boxplots of the fall velocity measured for each diameter class of the two disdrometers are plotted, distinguishing between rain events (a,b,d,e) and snow events (c,f). The plots provide qualitative information on the trend of the terminal fall velocity as a function of diameter: the median is plotted in red for LPM (bottom panels) and in green for 3DS (top panels); the first and third quartiles correspond to the lower and upper extremes of each box; the vertical dotted lines are delimited by the minimum and the maximum velocity for the corresponding diameter class (excluding any outliers). For rain events, the (3) velocity size relation is also plotted. Up to a drop diameter of about 3 mm, there is an agreement between the median velocity with (3); above this threshold, an underestimation of the data by both instruments can be observed. A recent paper has explained the underestimation of velocity via an artifact of the low-level processing [40]. However, the results are obtained with a different disdrometer and cannot be straightforwardly extended to the LPM or 3DS. As a reference for snow events, the size–velocity relation for rimed dendrites from [41] (hereinafter LH) is used. It is worth noting that the GK and (3) are validated and accepted as a reference for rain, while many size–velocity relations have been proposed for different ice particles (e.g., [41], Table 1).

Filtering methods based on the reference terminal fall velocity of raindrops provided by Formula (3) are often applied in the literature, in particular for liquid precipitation, in order to remove spurious drops generated mainly from splashing of drops on the disdrometer’s structure and from wind effects. Therefore, raindrops whose velocity differs more than ±50% from GK are removed. The middle panels in Figure 4 show statistics on terminal fall velocity with respect to diameter classes in the case of rain events with filtered data. For large diameters, the filtered data of LPM are in better agreement with the reference curve (3), although an underestimation with respect to it is still evident. For 3DS, filtered data differ from original ones only for small diameters. A slight underestimation is present in both cases for larger diameters.

### 5.3. Detection Capability

**Figure 5:**
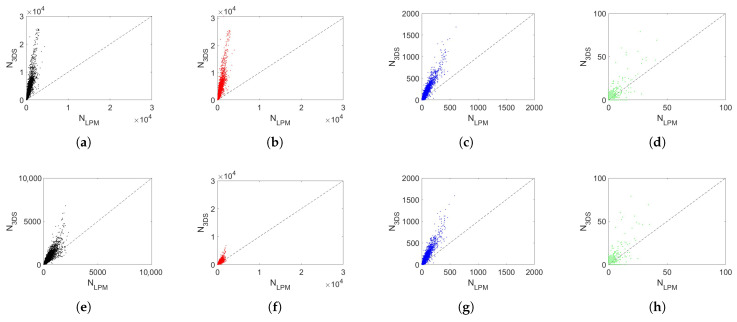
Scatterplot between the number of particles detected by the LPM (*x*-axis) and by the 3DS (*y*-axis) during rain events for (**a**,**e**) all raindrops; (**b**,**f**) small raindrops (D < 0.5 mm); (**c**,**g**) medium raindrops (0.5 mm < D < 1.5 mm); (**d**,**h**) large raindrops (D > 1.5 mm). Top panels refer to raw measurements, the bottom panels to filtered measurements.

Further analysis performed from spectrograms enables comparing the number of particles detected by the two disdrometers during rain events. Figure 5 shows the scatterplots between the number of particles detected by the LPM (*x*-axis) and those detected by the 3DS (*y*-axis). The comparison is completed for both unfiltered and filtered data (according to the criterion of censoring the 50% deviation from the reference curve (3)) and provides correlation values equal to 0.87 and 0.88, respectively. Moreover, 3DS detects more particles than LPM, probably due to the different measurement areas, although a more precise explanation would require more information on 3DS’s algorithms. Furthermore, the tendency of the 3DS disdrometer to record more particles than the LPM is greater for small diameters and tends to decrease significantly as droplet size increases. The 3DS detects 502% of small particles detected by LPM, 133% of medium particles, and −4% for large particles in the case of unfiltered data, providing an overall 468% irrespective of the diameters. If data filtering is applied, the number is reduced to 64%. The slope of the fit line is 6.1 for small diameters, 2.4 for medium diameters, and 1.3 for large diameters regarding unfiltered data. Since the measurement area of the 3DS is about three times that of the LPM, the slope of the medium droplets is in good relation to the ratio between the areas, while, for small and large hydrometeors, there is no relation between slope and the ratio of measurement areas. The application of the filter involves a clear decrease in slope in the case of small particles, reaching a value of 1.5, while for medium and large particles there are no major changes.

**Figure 6:**
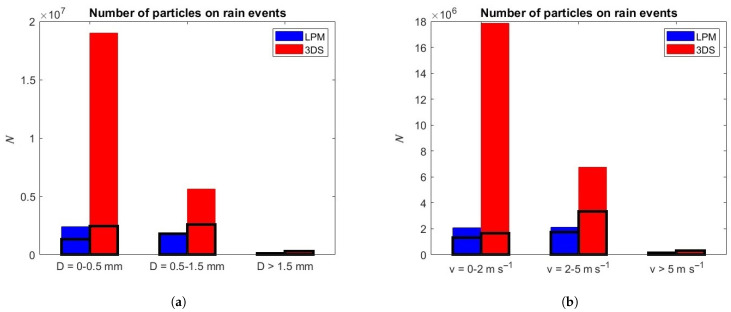
Barplot of the number of particles (*N*) detected by LPM and 3DS during rain events as a function of (**a**) diameter; (**b**) terminal fall velocity. Black lines mark the filtered values.

Finally, Figure 6 illustrates the barplot that highlights the differences between the two disdrometers in terms of number of detected particles during rain events as a function of the diameter (right) and fall velocity (left) of the hydrometeors. The main differences between 3DS and LPM occur for small diameters and low fall velocities. In particular, 3DS detects a higher number of small drops and a higher number of drops with low fall velocity.

Concerning the detection capabilities of minutes with rain of 3DS with respect to LPM, Table 3 shows the contingency table obtained considering all the minutes within rain events. The related statistical indices POD, FAR, and ACC are shown in Table 4. The number of hits minutes is very high compared to the false alarm, missing, or reject values. It is worth noting that, in the case of missing and false alarm minutes, the instrument that detects precipitation registers very low rain rate values: the average of the rain rate values detected by 3DS during false alarm minutes is 0.16 mm·h^−1^, while the average of the rain rate values detected by LPM during missing minutes is 0.17 mm·h^−1^. Consequently, the POD index value is equal to 99% and the relative accuracy is almost optimal.

### 5.4. Rainfall Rate

**Figure 7:**
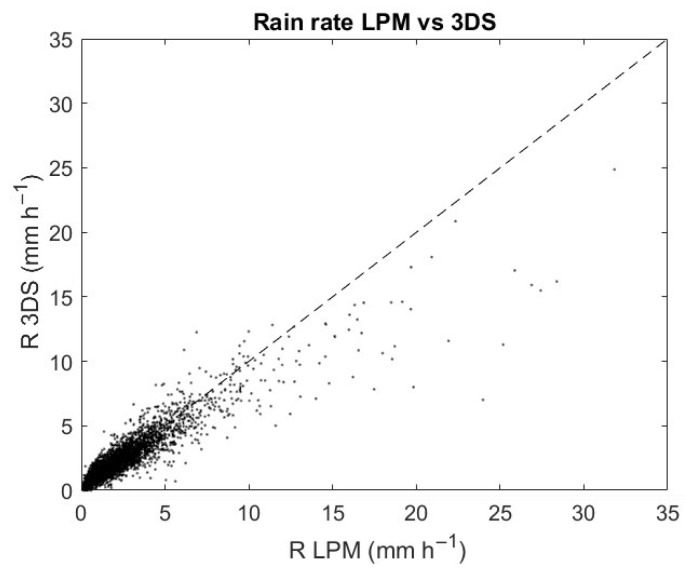
Scatterplot of rain rate detected by LPM (*x*-axis) and by 3DS (*y*-axis) during the common minutes of rain events from 13 December 2022 to 19 July 2023.

The final step deals with the calculation of the accumulated precipitation for each rain event, which is evaluated by using the rainfall rate values provided by the Thies Clima software. The 3DS provides two different precipitation intensity values per minute, one of which is ‘corrected’ by a multiplication factor indicated in the telegram. In this work, non-correct data are considered since the difference is minimal in terms of total cumulative precipitation per event: the correlation coefficient is practically equal to 1, while other indices (NMAE, RMSE, and NB) are very close to 0. Figure 7 shows the scatterplot of the precipitation rate in mm·h^−1^ estimated by LPM and 3DS during the common minutes included in the rain events of the validation campaign. In most of the rainy minutes, the rain rates are below 10 mm·h^−1^. For higher rain rates, the scatterplot shows a relative underestimation by 3DS. This is also confirmed by the values of maximum rain rate per event reported in Table A5 for the two disdrometers. Table 5 shows the values of the correlation coefficient RMSE (in mm·h^−1^), NMAE (in %), and NB (in %) between the accumulated precipitation values measured by the two devices.

Finally, the 3DS-based accumulated precipitation obtained in each event is compared with that obtained from the LPM.

**Figure 8:**
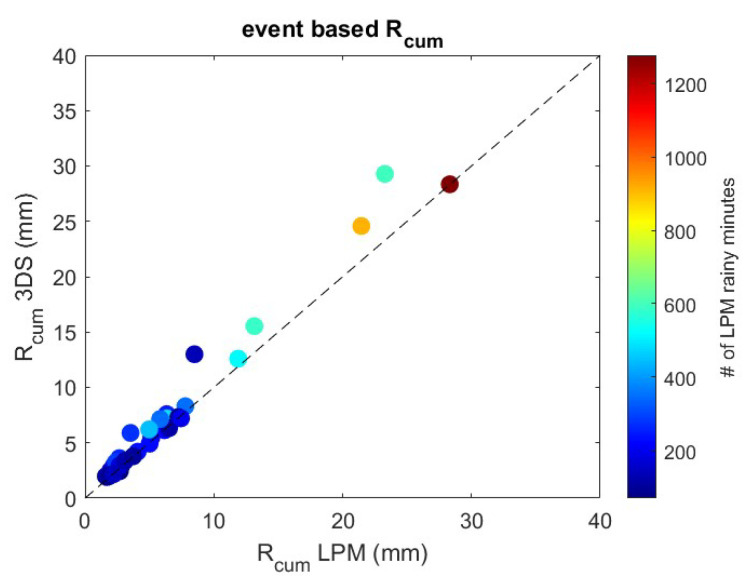
Scatterplot between event-based rainfall accumulation via LPM (*x*-axis) and via 3DS (*y*-axis).

Figure 8 shows the scatterplot between the event-based rainfall accumulation estimated by LPM (*x*-axis) and 3DS (*y*-axis). The color bar represents the number of rainy minutes collected by the LPM during the event. Table 6 provides the same factors of Table 5 but between precipitation accumulated in the 37 rainy events measured by the disdrometers. The agreement between the measurements of the two instruments is also excellent for long-lasting events and/or events with large cumulative rainfall. Moreover, the difference in the number of drops detected does not affect the final values of the cumulative precipitation per event.

It is worth noting that the 3DS sensor overestimates the number of detected hydrometeors compared to the LPM, reaching a small overestimation of the accumulated rainfall, as shown in Figure 8 and Table 6 (i.e., positive NB values). An overall overestimation of the 3DS-based 1 min rainfall rate with respect to the LPM is shown also in Figure 7 and Table 5. In this case, we notice that, for higher rainfall rate (i.e., R > 10 mm·h^−1^), 3DS underestimates the rainfall rate with respect to LPM. It should be reminded that, in this paper, we have used the values of rainfall rate provided by the manufacturer software. The 3DS manual does not provide a description of how the rainfall rate is calculated and it is not easy to infer the relation between the number of particles detected by 3DS and the corresponding rain rate. Furthermore, no information is provided on how to obtain DSD from the occurrence of drops provided by the 3DS software, and therefore this document does not report any comparison between the two disdrometers in terms of DSD.

### 5.5. Imaging Precipitation Measurements by 3DS

**Figure 9:**
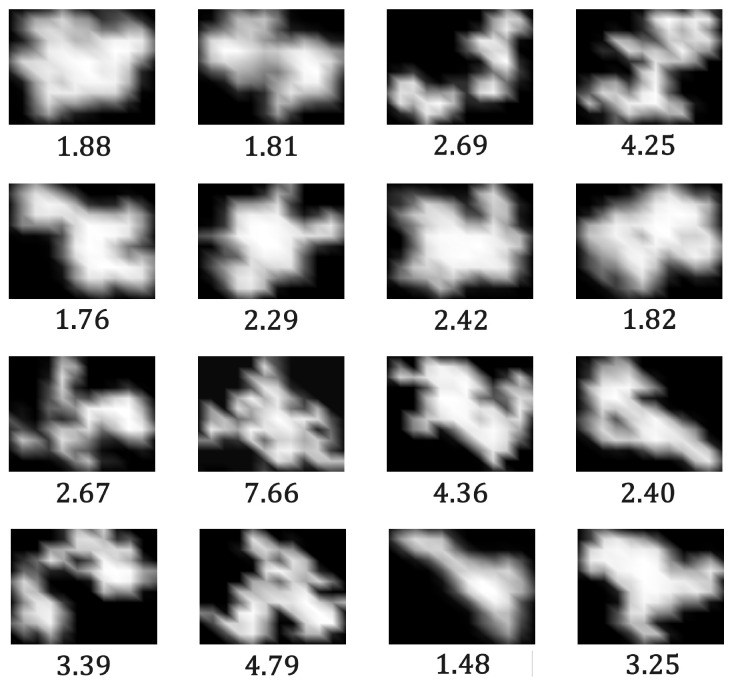
Images of particles obtained by 3DS disdrometer during snow events, classified as ‘snow’. The diameter (in mm) is provided at the bottom of each image.

**Figure 10:**
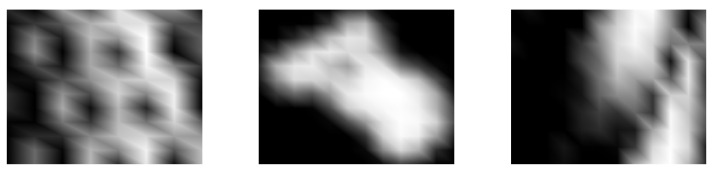
Sequence of images referring to 2023-01-20 19:00, classified as ‘rain with snow’ by the 3DS.

The possibility of recording images of precipitating particles is among the most attractive features of the 3DS disdrometer. The images captured during snow events are particularly interesting. Figure 9 shows some particles detected by the 3DS during the snow events of the validation campaign and classified by the instrument as ‘snow’. Information about the size (in mm) of these snowflakes is provided at the bottom of each image. These images refer to distinct minutes within the snow events. The 3DS disdrometer can detect multiple images per minute, but generally not all of them provide a good representation of the hydrometeor (see Figure 10).

**Figure 11:**
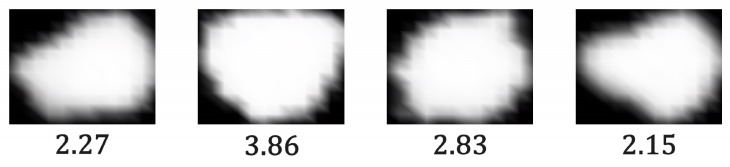
Images of particles obtained by 3DS disdrometer during hail minutes, classified as ‘hail’. The diameter (in mm) is provided at the bottom of each image.

The images in Figure 11 refer to minutes classified as hail by the 3DS. Again, information about the size of these particles is provided at the bottom of each image.

**Figure 12:**
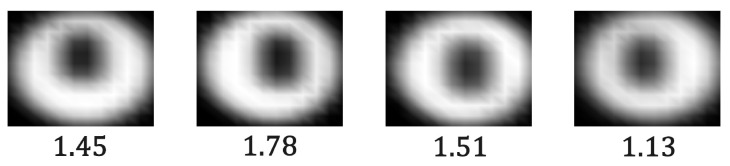
Images of particles obtained by 3DS disdrometer during rain events, classified as ‘rain’. The diameter (in mm) is provided at the bottom of each image.

Figure 12 provides images of hydrometeors classified as ‘rain’, which have a different appearance than snow and hail particles. Information about the size of these raindrops is provided at the bottom of each image. The black hole in the raindrop images is an artifact due to the refractive index of water.

**Figure 13:**
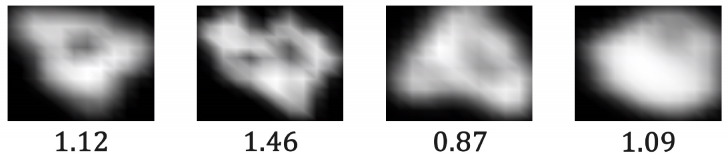
Images of particles obtained by 3DS disdrometer during minutes classified as ‘rain/drizzle with snow’. The diameter (in mm) is provided at the bottom of each image.

Finally, Figure 13 shows images of particles classified as ‘rain/drizzle with snow’ by the 3DS. The shape of these particles differs from the oblate shape that characterizes raindrops, but it is possible to notice the presence of a black hole that is also typical of hydrometeors classified as ‘rain’ in Figure 12 and can be representative of melting hydrometeors.

## 6. Conclusions

Imaging disdrometers have become quite popular in field campaigns, but, also due to their price, higher than that of laser disdrometers, their application is limited to research environments. Thies Clima has applied the concept of imaging disdrometers to a more affordable off-the-shelf product, named 3D stereo disdrometer. This paper has compared this instrument with the laser disdrometer of the same manufacturer (the Laser Precipitation Monitor). LPM can be considered a known instrument, being used in many research papers, while 3DS is a new device and, in the literature, there are few papers that deal with its data. The data used for the comparison were collected at a site in the Appennine range in Central Italy and report mostly rain precipitation. The two instruments are compared based only on outputs provided by the software of the manufacturer. In general, the 3DS detects more particles than the LPM, especially those with smaller diameters. This behavior is probably due to the different sampling modes of the two disdrometers and is different depending on diameters of drops. The 3DS in rain reports a lower percentage of superterminal drops than LPM. Both the disdrometers are in agreement with the reference size–velocity relation for rain drops with diameter less than 3 mm, while, for larger particles, underestimation of fall velocity occurs. The underestimation seems to be more pronounced for LPM, and the variability among the estimates is more spread around the median value. The two disdrometers agree quite well with each other in terms of rain rate and accumulated precipitation, although 3DS underestimates high rain rates with respect to LPM. The imaging capabilities of 3DS are of high interest in particular for identification and characterization of snow, ice, and mixed precipitation. However, to make such images useful for research, an improvement in the instruments is recommended. In fact, for the moment, the software provides the user with only a limited sample (from one to four particles per minute) of rather low-resolution images (12 × 12 pixel matrix) regardless of the dimensions of the hydrometeors). More images that show all (or a significant portion of) the particles that pass through the measuring area each minute can be useful and of high interest for precipitation microphysics-related studies. Improvement regarding the resolution of the images made available to the user is also recommended.

## Figures and Tables

**Table 1:** Contingency tables for detection analysis dealing with classification of LPM with respect to 3DS.

Class	Hits [min]	False Alarm [min]	Missing [min]	Reject [min]
Rain (or drizzle)	24,001	140	156	1926
Rain (or drizzle) with snow	42	31	528	25,622
Snow	1295	562	133	24,233
Hail	11	129	55	26,028

**Table 2:** Statistical indices obtained by contingency tables as in Table 1.

Class	POD [%]	FAR [%]	ACC [%]
Rain (or drizzle)	99%	1%	99%
Rain (or drizzle) with snow	7%	42%	98%
Snow	91%	30%	97%
Hail	17%	92%	99%

**Table 3:** Contingency table processed on all rain events for detection analysis of 3DS with respect to LPM disdrometer.

Hits [min]	False Alarm [min]	Missing [min]	Reject [min]
10,111	764	62	1839

**Table 4:** Statistical indices obtained by contingency tables of 3DS with respect to LPM disdrometer.

POD [%]	FAR [%]	ACC [%]
99	1	94

**Table 5:** Performance of the 3DS in estimating rainfall rate with respect to LPM.

Correlation	RMSE [mm·h^−1^]	NMAE [%]	NB [%]
0.93	0.7	24	6

**Table 6:** Performance of the 3DS in estimating rainfall accumulation with respect to LPM.

Correlation	RMSE [mm]	NMAE [%]	NB [%]
0.99	1.5	12	13

## Data Availability

3DS data are publicly available at https://zenodo.org/records/10258068 (accessed on 22 February 2024), while metadata are accessible through the Italian National Antarctic Data Center at https://antarcticdatacenter.cnr.it/geonetwork/srv/eng/catalog.search#/metadata/27e2bd39-097e-4512-96f0-fb213cd59a00 (accessed on 22 February 2024). LPM data will be published, along with the CORE-LAQ data, at the end of the campaign.

## Appendix A

Table A1 and Table A2 show the bin classes for diameters of LPM and 3DS, respectively. Table A3 and Table A4 show the bin classes for fall velocities of LPM and 3DS, respectively.

**Table A1:** Laser Precipitation Monitor class binning diameter.

LPM Class	Lower Limit [mm]	Class Width [mm]
1	0.125	0.125
2	0.250	0.125
3	0.375	0.125
4	0.500	0.250
5	0.750	0.250
6	1.000	0.250
7	1.250	0.250
8	1.500	0.250
9	1.750	0.250
10	2.000	0.500
11	2.500	0.500
12	3.000	0.500
13	3.500	0.500
14	4.000	0.500
15	4.500	0.500
16	5.000	0.500
17	5.500	0.500
18	6.000	0.500
19	6.500	0.500
20	7.000	0.500
21	7.500	0.500
22	8.000	∞

**Table A2:** 3D stereo class binning diameter.

3DS Class	Lower Limit [mm]	Class Width [mm]
1	0.000	0.100
2	0.100	0.100
3	0.200	0.100
4	0.300	0.100
5	0.400	0.100
6	0.500	0.200
7	0.700	0.200
8	0.900	0.200
9	1.100	0.200
10	1.300	0.200
11	1.500	0.400
12	1.900	0.400
13	2.300	0.400
14	2.700	0.400
15	3.100	0.400
16	3.500	0.800
17	4.300	0.800
18	5.100	0.800
19	5.900	0.800
20	6.700	0.800
21	7.500	1.600
22	9.100	3.200

**Table A3:** Laser Precipitation Monitor class binning velocity.

LPM Class	Lower Limit [m$\cdot s^{-1}$]	Class Width [m$\cdot s^{-1}$]
1	0.000	0.200
2	0.200	0.200
3	0.400	0.200
4	0.600	0.200
5	0.800	0.200
6	1.000	0.400
7	1.400	0.400
8	1.800	0.400
9	2.200	0.400
10	2.600	0.400
11	3.000	0.400
12	3.400	0.800
13	4.200	0.800
14	5.000	0.800
15	5.800	0.800
16	6.600	0.800
17	7.400	0.800
18	8.200	0.800
19	9.000	1.000
20	10.000	10.000

**Table A4:** 3D stereo class binning velocity.

3DS Class	Lower Limit [m$\cdot s^{-1}$]	Class Width [m$\cdot s^{-1}$]
1	0.000	0.200
2	0.200	0.200
3	0.400	0.200
4	0.600	0.200
5	0.800	0.400
6	1.200	0.400
7	1.600	0.400
8	2.000	0.400
9	2.400	0.800
10	3.200	0.800
11	4.000	0.800
12	4.800	0.800
13	5.600	1.600
14	7.200	1.600
15	8.800	1.600
16	10.400	1.600
17	12.000	3.200
18	15.200	3.200
19	18.400	3.200
20	21.600	6.400

## Appendix B

Table A5 shows information about the precipitation rate during rain events. For each event *N*, the duration (in minutes) and the minimum, maximum, and average rain rate values (in mm·h^−1^) recorded by the two disdrometers are reported.

**Table A5:** Duration and rain rate information per event.

N	Duration [min]	R_*min*_ LPM [mm·h^−1^]	R_*min*_ 3DS [mm·h^−1^]	R_*max*_ LPM [mm·h^−1^]	R_*max*_ 3DS [mm·h^−1^]	R_*mean*_ LPM [mm·h^−1^]	R_*mean*_ 3DS [mm·h^−1^]
1	720	0.1	0.1	10.7	11.6	2.3	2.8
2	235	0.1	0.1	4.2	3.4	0.9	1.0
3	346	0.1	0.1	6.9	6.8	1.7	1.7
4	1058	0.1	0.1	9.5	9.8	1.4	1.6
5	819	0.1	0.1	7.9	5.5	1.3	1.5
6	585	0.1	0.1	8.8	6.4	1.4	1.5
7	331	0.1	0.1	16.0	13.6	1.7	1.7
8	314	0.1	0.1	12.1	9.6	1.4	1.5
9	322	0.1	0.1	9.1	9.0	1.5	1.7
10	730	0.1	0.1	4.6	6.1	0.8	0.9
11	117	0.1	0.2	6.3	6.3	2.1	2.2
12	136	0.1	0.2	2.8	3.0	1.0	1.1
13	349	0.1	0.1	3.5	3.0	0.6	0.8
14	291	0.1	0.1	5.4	5.2	0.7	0.9
15	139	0.1	0.1	19.8	11.2	2.3	1.9
16	339	0.1	0.1	3.0	2.5	0.5	0.7
17	133	0.1	0.1	24.0	12.0	2.3	1.8
18	141	0.1	0.1	3.5	3.5	1.0	1.1
19	161	0.1	0.1	16.2	9.5	3.4	3.3
20	90	0.4	0.3	4.1	2.8	1.4	1.3
21	369	0.1	0.1	2.8	2.7	0.8	0.9
22	251	0.1	0.1	7.2	4.8	0.9	1.0
23	281	0.1	0.1	4.3	3.1	1.0	1.0
24	139	0.1	0.2	4.0	3.7	1.6	1.7
25	376	0.1	0.1	3.8	3.9	1.0	1.2
26	442	0.1	0.1	8.9	8.3	1.3	1.4
27	151	0.1	0.1	8.3	6.2	2.3	2.3
28	118	0.2	0.2	31.8	24.9	5.8	4.5
29	1382	0.1	0.1	7.2	5.7	1.3	1.3
30	283	0.1	0.1	8.7	5.1	1.3	1.3
31	571	0.1	0.1	3.5	4.0	0.7	0.8
32	212	0.1	0.1	4.3	3.2	0.8	0.9
33	112	0.1	0.1	5.6	5.6	1.2	1.3
34	179	0.1	0.1	27.4	20.9	3.9	3.2
35	93	0.2	0.2	7.2	6.5	1.8	1.8
36	203	0.1	0.1	5.4	2.3	0.9	0.9
37	269	0.1	0.1	19.7	14.6	2.2	2.2
